# Learning Disabilities in Extremely Low Birth Weight Children and Neurodevelopmental Profiles at Preschool Age

**DOI:** 10.3389/fpsyg.2016.00998

**Published:** 2016-06-28

**Authors:** Chiara Squarza, Odoardo Picciolini, Laura Gardon, Maria L. Giannì, Alessandra Murru, Silvana Gangi, Ivan Cortinovis, Silvano Milani, Fabio Mosca

**Affiliations:** ^1^NICU, Department of Clinical Sciences and Community Health, Fondazione IRCCS Ca' Granda Ospedale Maggiore Policlinico, Università degli Studi di MilanoMilan, Italy; ^2^Laboratory of Medical Statistics, Biometry and Epidemiology, Department of Clinical Sciences and Community Health, Università degli Studi di MilanoMilan, Italy

**Keywords:** extremely low birth weight, Griffiths scales, neurodevelopmental profile, learning disabilities, school outcome

## Abstract

At school age extremely low birth weight (ELBW) and extremely low gestational age (ELGAN) children are more likely to show Learning Disabilities (LDs) and difficulties in emotional regulation. The aim of this study was to investigate the incidence of LDs at school age and to detect neurodevelopmental indicators of risk for LDs at preschool ages in a cohort of ELBW/ELGAN children with broadly average intelligence. All consecutively newborns 2001–2006 admitted to the same Institution entered the study. Inclusion criteria were BW < 1000 g and/or GA < 28 weeks. Exclusion criteria were severe cerebral injuries, neurosensory disabilities, genetic abnormalities, and/or a Developmental Quotient below normal limits (< 1 SD) at 6 years. The presence of learning disabilities at school age was investigated through a parent-report questionnaire at children's age range 9–10 years. Neurodevelopmental profiles were assessed through the Griffiths Mental Development Scales at 1 and 2 years of corrected age and at 3, 4, 5, and 6 years of chronological age and were analyzed comparing two groups of children: those with LDs and those without. At school age 24 on 102 (23.5%) of our ELBW/ELGAN children met criteria for LDs in one or more areas, with 70.8% comorbidity with emotional/attention difficulties. Children with LDs scored significantly lower in the Griffiths Locomotor and Language subscales at 2 years of corrected age and in the Personal-social, Performance and Practical Reasoning subscales at 5 years of chronological age. Our findings suggest that, among the early developmental indicators of adverse school outcome, there is a poor motor experimentation, language delay, and personal-social immaturity. Cognitive rigidity and poor ability to manage practical situations also affect academic attainment. Timely detection of these early indicators of risk is crucial to assist the transition to school.

## Introduction

Survival rates of preterm infants, especially with extremely low birth weight (ELBW) and/or extremely low gestational age at birth (ELBW and/or ELGAN, respectively), have steadily increased in the last decades due to the improvement of medical knowledge and techniques used in intensive care (Doyle et al., 2011; Latini et al., 2013). As a consequence, the incidence of major sequelae has decreased whereas an increased risk of minor neurobehavioral and cognitive long-term deficits has been reported (Marlow et al., 2005).

Studies on the neuropsychological outcome in ELBW/ELGAN children have disclosed impairments across a wide range of areas, including planning and organization, self-regulation, mental flexibility, and deployment of attention (Farooqi et al., 2013). Difficulties in these areas have been demonstrated to be strongly associated with academic struggles and higher rates of special education support (Msall, 2012).

Consequently, at school age ELBW and/or ELGAN children are more likely to show poorer academic performance than their peers (Litt et al., 2012; Simms et al., 2013). In addition, several authors have found a high rate of Learning Disabilities (LDs) and difficulties in emotional regulation in very and extremely preterm children (Aarnoudse-Moens et al., 2009; Taylor et al., 2011; Lobo and Galloway, 2013). Even ELBW children with IQ scores within broadly normal limits frequently experience difficulties in academic achievement and attention (Grunau et al., 2002).

The identification of early developmental markers that may be predictive of school outcome is essential to provide timely interventions and improve cognitive abilities (Orton et al., 2009). While most studies have investigated neonatal factors that may predict long term school outcomes (Johnson et al., 2011), few have focused on the association between early neurodevelopmental markers that can be identified using the assessment tools commonly administered in clinical and research practice and learning difficulties at school age. Indeed, the general quotients obtained at 2 and 3 years of age with the Griffiths Mental Development Scales were found to strongly correlate with intellectual ability at 5 years assessed by the Stanford Binet in a cohort of ELBW infants (Bowen et al., 1996) and moderately correlate with the Wechsler Preschool and Primary Scale for Intelligence-Revised (WPPSI-R) in a study including infants born at term and with normal birth-weight (Sutcliffe et al., 2010).

The aim of this study was to investigate the incidence of LDs at school age (age range: 9–10 years) in a cohort of children born with ELBW and/or ELGAN with broadly average intelligence and to analyse their neurodevelopmental profiles at preschool age, in order to identify early indicators of risk for adverse school outcomes. Secondly, the prevalence of attention and/or emotional difficulties at school age was explored.

## Materials and methods

### Study design and participants

We performed a single-center longitudinal cohort study. The study was approved by the Ethics Committee of the Fondazione IRCCS Ca' Granda Ospedale Maggiore Policlinico and written informed consent was obtained from all parents.

Inclusion criteria were having a birth weight between 401 and 1000 g at birth (ELBW) and/or being born between 22 and 27^+6^ weeks gestation (ELGAN). Exclusion criteria were the presence of severe cerebral injuries [defined as intraventricular hemorrhage (IVH) grade 3–4 and/or periventricular lekomalacia (PVL) grade 2–4], neurosensory disabilities (blindness, deafness), genetic abnormalities and/or a Developmental Quotient assessed using the Griffiths Mental Development Scales Extended Revised (GMDS-ER) below normal limits (General Quotient < 1 SD) at 6 years.

**Figure 1:**
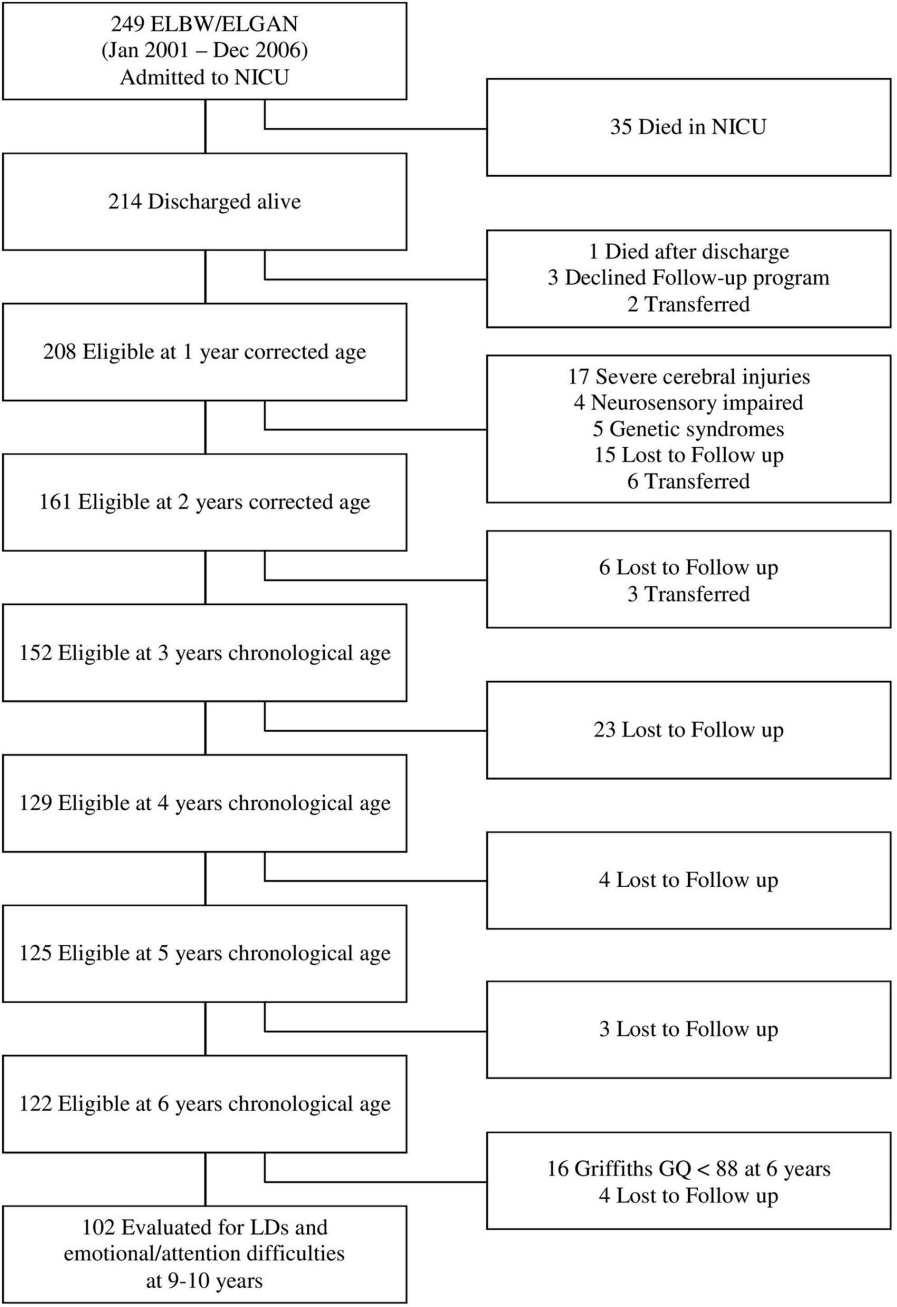
**Flow chart of the study**.

The flow chart of the study is shown in Figure 1. Of all the 249 ELBW and/or ELGAN infants admitted to NICU Fondazione IRCCS Ca' Granda Ospedale Maggiore Policlinico, between 2001 and 2006, 214 (85.9%) were discharged home alive and enrolled in the Follow-up program provided by the Authors' Institution. Of these, 122 (57.0%) returned for the 6 years follow-up visit and 102 (47.7%) entered the study.

### Procedure

Infants were enrolled at 1 year of corrected age and were prospectively followed up to age of 9–10 years. On the basis of the school outcome, children were divided into two groups: those with a LD diagnosis (LD-Group) and those without (No-LD-Group).

To investigate the presence of neurodevelopmental indicators of risk for LDs, the neurodevelopmental outcomes at 1 and 2 years of corrected age and at 3, 4, 5, and 6 years of chronological age were analyzed comparing two groups of children on the basis of the presence or not of the LD diagnosis.

#### Collection of perinatal and social characteristics

Basic subjects' characteristics (gender, birth weight, being adequate or small for gestational age, mode of delivery, multiple birth, duration of hospital stay, number of days on mechanical ventilation) were collected from the infants' computerized medical charts. Gestational age was based on the last menstrual period and early ultrasound examination; infants with birth weight ≥10th centile or < 10th centile for gestational age, according to the Fenton Growth Chart (Fenton, 2003), were classified respectively as Adequate or Small for Gestational Age (AGA/SGA). The occurrence of sepsis, necrotizing enterocolitis (NEC) of stage 2 or higher, according to the classification of Bell et al. (1978), intraventricular hemorrhage (IVH) grade 3 or higher, according to the Papile classification scheme (Papile et al., 1983), periventricular leukomalacia (PVL) of grade 2 or higher, according to de Vries et al. (1992), retinopathy of prematurity (ROP) of stage 3 or higher, according to the (Committee for the Classification of Retinopathy of Prematurity, 1984), and bronchopulmonary dysplasia (BPD), defined as oxygen supplementation at 36 weeks postmenstrual age (Jobe and Bancalari, 2001), were also collected. Sepsis was defined by the presence of positive blood and/or cerebrospinal fluid culture. IVH and PVL were detected by brain magnetic resonance imaging examination at 40 weeks postmenstrual age. Corrected age was calculated up to 24 months of life, from the chronological age adjusting for gestational age. Mothers' nationality and education were also recorded. Mothers' educational level was used as a measure of socioeconomic status and classified using a 3 point scale, where 1 indicates primary or intermediate school education (≤ 8 years), 2 secondary school education (9–13 years) and 3 university degree (>13 years).

#### Screening of LDs and emotional/attention difficulties at school age

**Figure 2:**
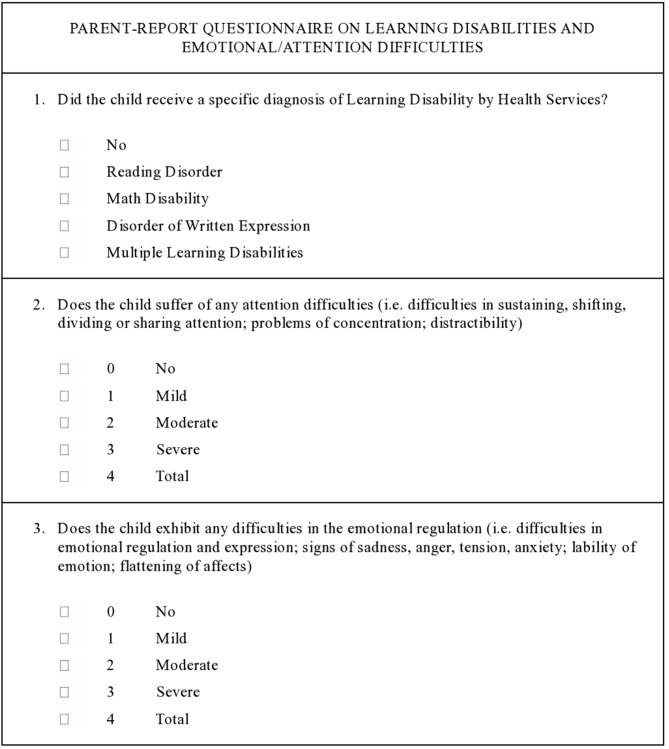
**Parent-report questionnaire on Learning Disabilities and emotional/attention difficulties at school age**.

In order to screen the rates of academic difficulties in our sample and the possible co-occurrence of attention and/or emotional problems a parent-report questionnaire was developed (Figure 2). Before starting the study, the questionnaire was tested with a sample of parents to clarify any doubts on item comprehension.

The questionnaire was announced by phone to the parents, when their infants were aged 9–10, by an expert psychologist that accurately explained each question and response option. All questionnaires were sent by post and parents were asked to fill-in and return them the same way.

The presence of a Learning Disability was labeled if the child had a certificated diagnosis of Learning Disability, provided by Health Services and based on the International Statistical Classification of Diseases and Related Health Problems, 10th Revision (ICD-10) criteria (WHO, 2010). Specifically, Learning Disabilities were defined as a specific and significant impairment in the development of reading/writing/computing skills, in children without neurosensory impairment or inadequate schooling, who had estimated normal IQs.

The presence of attention deficits and/or emotional disregulation, based respectively on the International Classification of Functioning, Disability and Health: Children and Youth Version (ICF-CY; WHO, 2007) definitions of Attention functions (code b140) and Emotional functions (code b152) was also investigated.

#### Neurodevelopmental assessment at preschool age

According to our Follow-up programme, neurodevelopmental outcome was assessed by means of the Griffiths Mental Development Scales Revised (GMDS-R; ages 0–2 years) and Extended Revised (GMDS-ER; ages 2–8 years) respectively (Griffiths and Huntley, 1996; Luiz et al., 2006), administered by two trained examiners.

The Griffiths comprises a set of 5 subscales (6 for the extended version), with composite scores ranging from 50 to 150. The Locomotor subscale allows the examiner to assess the child's gross motor skills. The Personal-Social subscale assesses the child proficiency in his activities of daily living, his levels of independence, as well as his ability to interact with other children. The Language subscale investigates both receptive and expressive verbal skills. The Eye and Hand Coordination subscale assesses the child's fine motor skills, visual monitoring skills, and manual dexterity. The Performance subscale investigates visuospatial skills including speed of working and precision. The Practical Reasoning subscale covers a range of skills that involve practical reasoning, such as learning numerical concepts and orientating in time and space.

The Scale yields standardized scores for each domain (mean 100, SD 16) and a composite General Quotient (mean 100, SD 12). Because normative data of the Griffiths Mental Development Scales Revised and Extended Revised are not available in our country, we referred to the 1996 and 2006 UK norms, respectively. The Italian-validated translation of the administration manuals have been used.

### Statistical analyses

The homogeneity between the two groups of children (with and without Learning Disabilities) has been verified through the confidence interval at 95% of the differences between either the means or the frequencies of the variables taken into consideration. The evolution of Griffiths scores (General Quotients and separately for each domain) was fitted with a general mixed model where age, outcome, and interaction age^*^outcome were introduced as fixed effect terms and child as random term. Results were expressed as least squares means (± standard error) and differences between groups as differences between least squares means (with 95% confidence interval of differences).

## Results

### Description of the study sample

Maternal and infants' basic characteristics are shown in Table 1.

**Table 1:** **Maternal and infant characteristics**.

**Characteristics**	**No LD group (*n* = 78)**	**LD group (*n* = 24)**	**C.I. 95% of differences**
**MATERNAL**			
Age, years (mean)	33.7	33.6	−2.0; 2.1
University degree, %	26.2	20.8	−14.4; 29.2
Italian nationality, %	82.0	79.2	−18.2; 23.9
**INFANT**			
Birth weight, g (mean)	813.0	804.0	−74.0; 91.0
Gestational age, weeks (mean)	27.8	27.7	−0.9; 1.0
Males, %	38.5	50.0	−13.9; 37.0
SGA, %	52.6	50.0	−23.0; 28.2
Multiple birth, %	17.9	16.7	−18.6; 21.2
Cesarean delivery, %	89.7	87.5	−15.3; 19.8
Sepsis, %	30.7	50.0	−5.8; 44.4
NEC stage 2–3, %	1.3	8.3	−7.0; 21.1
IVH grade 3–4, %	1.3	4.2	−8.2; 14.0
PVL, %	1.3	4.2	−8.2; 14.0
BPD, %	42.3	45.8	−21.9; 29.0
ROP grade 3–4, %	6.4	25.0	−2.3; 39.5
Days in hospital (mean)	88.6	98.5	−5.2; 25.0
Days on ventilation (mean)	9.1	12.4	−2.8; 9.4

As shown by confidence intervals, there were no significant differences between LD-Group and No-LD-Group for each of the variables considered. Nevertheless, LD-Group includes a slightly higher rate of males (though not reaching a statistical significance).

The mean ages at testing for both groups were respectively 12.3 ± 0.5 months and 23.9 ± 0.5 months of corrected age and 36.1 ± 0.5 months, 48.1 ± 0.5 months, 60.1 ± 0.5 months, and 72.1 ± 0.5 months of chronological age.

Although 18.0 and 20.8% of mothers in No-LD-Group and LD-Group respectively were not Italian, all infants attended a kindergarten or a preschool education programme and so were exposed to Italian as a primary language in their community environment.

### School outcomes

All parents who were telephonically contactable (96.0%) at child age 9–10 accepted to fill in the questionnaire on school outcome.

At school-age, 24 on 102 (23.5%) of the ELBW/ELGAN children with broadly normal intelligence (Griffiths General Quotient within ± 1 SD at 6 years) met criteria for LD in 1 or more areas. LDs in reading, written expression and arithmetic occurred respectively in 1.0, 3.9, and 4.9% of the whole study sample. Learning disabilities affecting more than 1 domain occurred in 13.7% of the children (Table 2).

**Table 2:** **LDs in reading, written expression and arithmetic in the whole sample and LD-group**.

	**Whole sample (*n* = 102) *N* (%)**	**LD-group (*n* = 24) *N* (%)**
No LDs	78 (76.5)	0 (0.0)
Reading	1 (1.0)	1 (4.2)
Written expression	4 (3.9)	4 (16.7)
Arithmetic	5 (4.9)	5 (20.8)
More than one	14 (13.7)	14 (58.3)
Total LDs	24 (23.5)	24 (100.0)

In the group who met criteria for LDs (LD-Group), only 29.2% children were free from emotional or attention difficulties, compared to 69.2% children in the No-LD-Group. The prevalent difficulty in the LD-Group was in the emotional area (25.0%) and secondary in the attention area (16.7%). It is relevant to note that 29.2% children reported a fragility in both areas (Table 3).

**Table 3:** **Emotional and/or attention difficulties in No-LD-group vs. LD-group**.

	**No-LD-group (*n* = 78) *N* (%)**	**LD-group (*n* = 24) *N* (%)**
No difficulties	54 (69.2)	7 (29.2)
Emotional difficulties (only)	12 (15.4)	6 (25.0)
Attention difficulties (only)	8 (10.3)	4 (16.7)
Both difficulties	4 (5.1)	7 (29.2)

### Preschool outcomes

Table 4 shows the differences in mean scores (estimated with a general mixed model) between LD-Group vs. No-LD-Group Griffiths' General Quotients and Subquotients at 1 and 2 years of corrected age, and at 3 to 6 years of chronological age.

**Table 4:** **Estimated Griffiths mean scores in No-LD-Group vs. LD-Group**.

	**LD-group (*n* = 78)**	**No-LD-group (*n* = 24)**	**Estimated Griffiths score differences**	
	**Estimated Mean (*S.E*.)**	**Estimated Mean (*S.E*.)**	**Mean score difference**	**95% C.I**.
**1 YEAR CORRECTED AGE**				
General Quotient	94.0 (1.2)	97.5 (1.2)	3.5	0.1 to 6.9
Scale A—Locomotor	88.5 (2.0)	92.5 (1.5)	4.0	−0.9 to 8.9
Scale B—Personal–Social	93.0 (1.3)	95.0 (1.1)	2.0	−1.4 to 5.3
Scale C—Language	98.1 (1.6)	101.4 (1.2)	3.3	−0.6 to 7.2
Scale D—Eye and Hand Coordination	91.2 (1.8)	97.0 (1.3)	5.8	1.3 to 10.2
Scale E—Performance	94.4 (1.5)	97.0 (1.4)	2.6	−1.6 to 6.7
**2 YEARS CORRECTED AGE**				
General Quotient	87.7 (2.9)	95.7 (1.3)	8.0	1.6 to 14.4
Scale A—Locomotor	89.9 (3.2)	98.6 (1.7)	8.7	1.4 to 15.9
Scale B—Personal–Social	83.0 (3.4)	90.3 (1.7)	7.3	−0.1 to 14.8
Scale C—Language	82.6 (3.5)	94.2 (1.8)	11.6	3.8 to 19.3
Scale D—Eye and Hand Coordination	96.5 (2.4)	100.1 (2.0)	3.6	−1.8 to 9.0
Scale E—Performance	91.0 (3.1)	95.5 (1.7)	4.5	−2.6 to 11.6
**3 YEARS CHRONOLOGICAL AGE**				
General Quotient	93.3 (1.3)	96.1 (0.9)	2.8	−0.3 to 5.9
Scale A—Locomotor	98.9 (1.7)	99.2 (1.1)	0.3	−3.7 to 4.2
Scale B—Personal–Social	94.4 (1.9)	96.9 (1.1)	2.5	−1.9 to 6.8
Scale C—Language	89.9 (2.5)	92.7 (1.5)	2.8	−3.0 to 8.6
Scale D—Eye and Hand Coordination	88.9 (1.4)	92.7 (0.9)	3.8	0.6 to 7.1
Scale E—Performance	98.7 (1.3)	100.0 (0.9)	1.3	−1.8 to 4.5
Scale F—Practical Reasoning	90.8 (1.9)	95.9 (1.0)	5.1	0.8 to 9.5
**4 YEARS CHRONOLOGICAL AGE**				
General Quotient	94.1 (1.0)	95.8 (0.8)	1.7	−0.8 to 4.2
Scale A—Locomotor	97.7 (0.8)	97.3 (1.1)	−0.4	−3.1 to 2.2
Scale B—Personal–Social	93.8 (1.4)	96.6 (1.1)	2.6	−0.8 to 6.1
Scale C—Language	96.6 (1.8)	97.5 (1.2)	0.9	−3.2 to 5.1
Scale D—Eye and Hand Coordination	88.6 (1.2)	92.8 (1.3)	4.2	0.7 to 7.7
Scale E—Performance	95.6 (1.0)	96.4 (0.8)	0.8	−1.8 to 3.4
Scale F—Practical Reasoning	94.2 (1.6)	94.0 (0.8)	−0.2	−3.9 to 3.4
**5 YEARS CHRONOLOGICAL AGE**				
General Quotient	93.5 (0.9)	96.3 (0.6)	2.8	0.8 to 4.9
Scale A—Locomotor	96.8 (1.3)	98.0 (0.7)	1.2	−1.8 to 4.2
Scale B—Personal–Social	93.7 (0.8)	96.7 (0.7)	3.0	0.9 to 5.1
Scale C—Language	95.1 (1.4)	97.6 (0.7)	2.5	−0.7 to 5.6
Scale D—Eye and Hand Coordination	94.8 (1.3)	97.6 (0.7)	2.8	−0.2 to 5.8
Scale E—Performance	91.5 (1.3)	97.0 (0.8)	5.5	2.5 to 8.5
Scale F—Practical Reasoning	88.8 (1.5)	92.8 (0.7)	4.0	0.7 to 7.3
**6 YEARS CHRONOLOGICAL AGE**				
General Quotient	95.9 (0.7)	98.1 (0.6)	2.2	0.3 to 4.0
Scale A—Locomotor	97.6 (1.1)	99.4 (0.7)	1.8	−0.6 to 4.4
Scale B—Personal–Social	95.5 (1.1)	97.8 (0.6)	2.3	−0.2 to 4.8
Scale C—Language	96.4 (0.6)	97.3 (0.6)	0.9	−0.7 to 2.5
Scale D—Eye and Hand Coordination	98.0 (0.8)	100.9 (0.7)	2.9	0.8 to 5.0
Scale E—Performance	95.7 (1.0)	99.2 (1.0)	3.5	0.7 to 6.3
Scale F—Practical Reasoning	92.1 (1.9)	93.3 (0.9)	1.2	−1.8 to 4.3

The mean General Quotients of the two groups show the highest difference at 2 years of corrected age (8.0 points of difference), with the LD Group standing between a low-average and a below-the-mean level. At later ages the discrepancy tends to decrease to 2.8 points (5 years) and 2.2 points (6 years), though remaining statistically significant. An overall improvement can be observed in the LD-Group General Quotients, while No-LD-Group mean scores remain more stable.

Considering the differences between the two groups' mean subquotients, the 2 and 5-years assessments are the most noteworthy regarding both the variety of neurodevelopmental domains and the wideness of the score differences. Conversely, at the other assessment stages the discrepancy between the two groups' subquotients is more subtle.

At 2 years of corrected age the largest gap between the two groups emerges in the Language (11.6 points of difference), and the Locomotor (8.7 points) subscales, while at 5 years of chronological age the widest differences are in the Performance (5.5 points), Practical Reasoning (4.0) and Personal-Social (3.0) subscales.

A quite stable significant difference in the Eye and Hand Coordination subscale emerges both at 1 year of corrected age (5.8 points of difference) and at 3 years (3.8 points), 4 years (4.2 points), and 6 years (2.9) of chronological age.

## Discussion

### Outcome at school age

Our study shows that 23.5% of our ELBW/ELGAN children with broadly normal intelligence met criteria for LD with a prevalence of Learning Disabilities in multiple domains (13.7%).

Attention and/or emotional difficulties were found in 70.8% of children with LDs, but only in 30.8% of children without LDs.

Compared to the last reports of the Italian Ministry of Education, University and Research referring to 2011/2012 academic years (MIUR, 2013), the incidence of LDs in our sample is significantly higher than the overall regional population (23.5 vs. 2.0%). This is confirmed by other authors, highlighting that ELBW children are more likely to show difficulties in basic academic skills and multiple learning domains compared to children born at term or with weight >2500 g (Salt and Redshaw, 2006; Wocadlo and Rieger, 2006).

Our rates of LDs are comparable to those of Johnson et al. (2011). The authors investigated the academic attainment at 11 years of a cohort of ELBW infants born < 26 weeks gestation free from severe neurosensory and cognitive impairment and found 27.0% LDs (vs. 23.5% of our study cohort). The high concordance with Johnson's findings is probably due to the exclusion of severely impaired children from their study, which was our main exclusion criteria too.

On the contrary, our rates are much lower than those reported by the EPICure study group (Johnson et al., 2009; 50.0%) and by Anderson and Doyle (2008; 36.0%). This difference may arise from the characteristics of the sample as these studies included extremely premature children (< 26 weeks Gestational age and/or < 750 g Birth weight) with severe neurosensitive and cognitive impairment (as previously defined) at 11 years.

### Neurodevelopmental profiles at preschool age

The neurodevelopmental profiles at preschool ages showed that the 2 years of corrected age and the 5 years of chronological age assessment stages were the most effective in discriminating between the LD-Group and No-LD-Group outcomes. Children with LDs actually showed significantly lower mean scores in Locomotor and Language subscales at 2 years of corrected age and both in Personal-Social and in Performance and Practical Reasoning subscales at 5 years of chronological age.

Early motor experimentation, enabling the child to discover the environment and take contact with objects and persons, enhances the maturation and organization of higher cognitive functions. Consequently, the low performance shown by the LD-Group in the Locomotor subscale at 2 years of corrected age might lead to a poorly integrated mental representation and organization of spatial experiences and objects. Indeed, in literature there is evidence of a strong association between early motor development and later intellectual functions within the normal population (Murray et al., 2006).

Besides, Walle and Campos (2014) demonstrated that walking onset is related to infant language development. Thus, we speculate that also the poor performance of the LD-Group in the Language subscale might be associated to a poor desire for experimentation, limiting the expansion of verbal expressive skills.

At 5 years of chronological age the LD-Group showed significantly lower scores in the Personal-Social, Performance and Practical Reasoning subscales.

For the LD-Group, the low scores in the Personal-Social subscale reveal a lack of personal independency and a poor motivation in differentiating from their caregivers.

The frequent co-occurrence of LDs and emotional deficits in our sample supports the hypothesis of an emotional immaturity in the LD-Group. Follow-up studies confirm that behavioral and socio-emotional impairments may negatively affect cognitive functions and academic achievements in preterms (Aarnoudse-Moens et al., 2009; Pugliese et al., 2013).

Since our sample included only children with broadly average intelligence, the discrepancy between the LD-Group and No-LD-Group in the Performance and Practical Reasoning Subquotients at 5 years seems not to be related to a cognitive impairment but mostly to a lack of flexibility in problem solving strategies and to a poor ability in managing everyday situations. This consistent with previous studies with very preterm or ELBW children, reporting impairments across a range of executive processes including planning and organizational ability, generation of new ideas and strategies and mental flexibility (Anderson et al., 2004).

As far as the Eye and Hand Coordination subscale is concerned, we speculate that the low mean scores shown by the LD-Group along all the study period may be related to an unsuitable coordination between eyes and hands but may also be mediated by an attention deficit. Several literature studies confirm that minor motor disabilities persist in survivors of preterm birth and that they often co-exist with behavioral deficits. Specifically, attention seems to be an area of specific weakness for preterm children (Foulder-Hughes and Cooke, 2003; Feder et al., 2005; Mulder et al., 2009). The high rate of attention difficulties reported at school age by this group of children strengthens our hypothesis.

## Limits of our study

The major limit of our study is the lack of a direct assessment of LDs at school age. Relying exclusively on the presence of a LD certification may raise doubts on the possible presence of undiagnosed LDs who didn't reach Health Services because not identified as suspected cases by teachers. However, the Italian legislation regarding Learning Disabilities requires a screening using standardized tests by the end of the second school year. Therefore, we are confident that all the children enrolled in our study who showed any academic difficulties have been correctly identified and addressed to specialists to receive a LD diagnosis.

## Conclusions

Our findings indicate that children born ELBW are particularly vulnerable to learning disabilities at school age associated with attention and emotional difficulties. Our findings suggest that among the early developmental indicators of adverse school outcome there is a poor motor experimentation, language delay and personal-social immaturity. The lack of cognitive flexibility and the poor ability to manage practical situations at preschool age also interfere with intellectual functioning, negatively affecting the academic attainment.

These results might be useful to established prevention and monitoring interventions and to facilitate the collaboration between the various figures involved in the child's care (healthcare professionals, teachers and families.

Focusing on these early indicators of risk for adverse school outcome is crucial to ensure an adequate support for the child, maximizing his/her abilities and assisting the transition to school.

## Author contributions

CS, LG, and MG conceptualized and designed the study, interpreted the clinical data of follow up, drafted the initial manuscript, and critically reviewed the manuscript. OP, AM, and SG designed the data collection instruments and critically reviewed the manuscript. IC and SM carried out the initial analyses, reviewed, and revised the manuscript. FM interpreted the clinical data of follow up and critically reviewed the manuscript. All authors read and approved the final manuscript and agree to be accountable for the content of the work.

### Conflict of interest statement

The authors declare that the research was conducted in the absence of any commercial or financial relationships that could be construed as a potential conflict of interest.

## Abbreviations

AGA/SGA: Adequate/Small for Gestational Age
BPD: Bronchopulmonary dysplasia
ELBW: Extremely Low Birth Weight
ELGAN: Extremely Low Gestational Age Newborns
IVH: Intraventricular hemorrhage
LD: Learning disability
NEC: Necrotizing enterocolitis
PVL: Periventricular leukomalacia
ROP: Retinopathy of prematurity.
